# Comparison of SpO_2_ and heart rate values on Apple Watch and conventional commercial oximeters devices in patients with lung disease

**DOI:** 10.1038/s41598-021-98453-3

**Published:** 2021-09-23

**Authors:** Leonardo Zumerkorn Pipek, Rafaela Farias Vidigal Nascimento, Milena Marques Pagliarelli Acencio, Lisete Ribeiro Teixeira

**Affiliations:** 1 Faculdade de Medicina FMUSP, Universidade de Sao Paulo, São Paulo, São Paulo Brazil; 2 Centro Universitário FMABC, Santo André, São Paulo Brazil; 3 Laboratorio de Pneumologia/LIM09-Divisao de Pneumologia, Instituto do Coracao (InCor), Hospital das Clinicas HCFMUSP, Faculdade de Medicina, Universidade de Sao Paulo, São Paulo, SP Brazil

**Keywords:** Respiratory tract diseases, Hypoxia

## Abstract

Lung diseases have high mortality and morbidity, with an important impact on quality of life. Hypoxemic patients are advised to use oxygen therapy to prolong their survival, but high oxygen saturation (SpO_2_) levels can also have negative effects. Pulse oximeters are the most common way to assess oxygen levels and guide medical treatment. This study aims to assess whether wearable devices can provide precise SpO_2_ measurements when compared to commercial pulse oximeters. This is a cross-section study with 100 patients with chronic obstructive pulmonary disease and interstitial lung disease from an outpatient pneumology clinic. SpO_2_ and heart rate data were collected with an Apple Watch Series 6 (Apple) and compared to two commercial pulse oximeters. The Bland–Altman method and interclass correlation coefficient were used to compare their values. We observed strong positive correlations between the Apple Watch device and commercial oximeters when evaluating heart rate measurements (r = 0.995, p < 0.001) and oximetry measurements (r = 0.81, p < 0.001). There was no statistical difference in the evaluation of skin color, wrist circumference, presence of wrist hair, and enamel nail for SpO_2_ and heart rate measurements in Apple Watch or commercial oximeter devices (p > 0.05). Apple Watch 6 is a reliable way to obtain heart rate and SpO_2_ in patients with lung diseases in a controlled environment.

## Introduction

In recent years, the Connected Health Model has gained great visibility. The term refers to the concept of using multiple resources, including wireless, digital, electronic, mobile, and telemedicine, for the benefit of patient's health. In this model, it is proposed that patients and health professionals are connected and sharing data, in order to improve care^1^.

In 2020, approximately 19% of Americans already used some wearable device^2^. Most of these devices were initially limited to collecting daily step data, but the great advances in technology have allowed other information to be collected effortlessly by users, including standing hours, movement, time and type of physical exercise, sleep tracking, heartbeat and, more recently, oxygen saturation.

The presence of all these data for the patient has already proved to be extremely effective as a way of promoting health. Frank et al. showed that sending reminders to users on these devices stimulating physical activity was able to change daily habits in students^3^. Other authors have also studied the advantages and limitations of wearable’s to prevent childhood obesity^4^ and weight loss^5^.

In addition to health promotion, these devices are also being tested to identify and diagnose medical conditions, including detection of falls^6^, mental status^7^, heart disease^8^, neurological diseases^9^ and others.

The latest version of Apple’s smartwatch (Apple Watch Series 6), a leading company in the technology sector, includes in its health monitoring capabilities the measurement of blood oxygen saturation. The continuous monitoring of this variable can bring important information to the care of patients with lung diseases.

Chronic obstructive pulmonary disease (COPD) is a health problem with a high prevalence and mortality, with approximately 5% of the world population affected in some degree^10^ and the fourth cause of death in the United States^11^. As part of the pathophysiological process of the disease, these patients have difficulty in gas exchange and decreased oxygen saturation. Dalbak et al. pointed out the use of pulse oximetry to assess the severity of the disease and assist in medical decision^12^.

In addition to clinical treatment in accordance with the guidelines established by Global Initiative for Chronic Obstructive Lung Disease (GOLD), hypoxemic patients are advised to use oxygen therapy to prolong their survival^13^. To obtain this benefit, these patients must use oxygen for at least 15 h a day and maintain an adequate flow. However, the increase in saturation should not exceed 92%, since the damage by additional oxidative stress may counteract the benefits of this therapy^14^.

A study with COPD patients identified that there is a great fluctuation in SpO_2_ during the day and from one day to the next^15^. Thus, punctual measures are not sufficient to predict the changes that occur in oxygen saturation and, therefore, guide the flow of oxygen that these patients must use at all times.

As in COPD, oxygen therapy is also instituted in most cases of patients with interstitial lung diseases (ILD)^16,17^. This is a group of lung diseases characterized by alveolar damage with fibroblastic and/or inflammatory proliferation. The natural course of the disease can lead to pulmonary fibrosis and, subsequently, respiratory failure. These diseases mostly affect connective tissue, undergoing respiratory restriction and hypoxemia, the latter being one of the main causes of evolution to respiratory failure^18^.

In this context, continuous monitoring of blood oxygen saturation can bring great benefits to patients using long-term oxygen therapy. Zhu et al. demonstrated that continuous monitoring in these patients increases the percentage of time they spend on the saturation target (88–92%) and decreases the amount of oxygen used by these patients^19^.

The objective of this study was to evaluate the precision of a wearable device (Apple Watch—Series 6 smartwatch, Apple, California) in an outpatient and controlled environment, by gathering data synchronously obtained from patients with two widely used conventional oximeter and the smartwatch. Our hypothesis is that the Apple Watch is a reliable method to measure SpO_2_ in patients with lung diseases under controlled circumstances.

## Methods

### Study approval

This research project was approved by the Ethics and Research Committee of the Hospital das Clinicas da Faculdade de Medicina da Universidade de Sao Paulo (HC-FMUSP). Online registration CAPPesq: 4.585.264 approved 11/March/2021 and on the Brazil platform CAAE number: 43161121.0.0000.0068. Apple was not involved in the design, implementation, data analysis, or manuscript preparation of the study.

All methods were performed in accordance with the relevant guidelines and regulations.

### Study design

This is a cross-section study with stable patients from the outpatient pneumology clinic of the HC-FMUSP. All patients and healthy individuals gave written informed consent.

Patients older than 18 years old with COPD or ILD were included in the study. A group of healthy volunteers was also included for comparison.

Patients unable to perform the necessary maneuvers to correctly measure oxygen saturation with the oximeter and smartwatch or clinically unstable were excluded from the study.

### Data collection

The following data were collected: age, gender, underlying disease, use of oxygen, skin color, wrist circumference and presence of hair at the wrist, digital clubbing and enamel nail. Oxygen saturation (SpO_2_) and heart rate were measured during data collection by a smartwatch and commercial oximeters.

The data mentioned above was collected during the interview with the patient and, if necessary, supplemented based on the electronic medical record.

The skin color was classified according to the Fitzpatrick scale.

The circumference of the wrist was measured using an anthropometric measuring tape.

The smartwatch used was an Apple Watch Series 6 (Apple), 44 mm, band small/medium and large. The commercial conventional oximeters used were Mobil POD-2 Finger Oximeter and Multilaser OX-06 Oximeter, both approved by ANVISA (National Health Surveillance Agency).

For the measurement of pulse oximetry and heartbeat, patients were properly seated in a comfortable position with the left arm resting on the table at the height of the heart. The Apple Watch was adjusted on the left wrist so that it does not become loose or tight, according to the instructions provided by the manufacturer. Commercial oximeters were placed on the index and middle fingers of the left hand.

Three measures were taken to measure oxygen saturation. The value displayed on the commercial oximeter at the exact moment the Apple Watch finish reading was noted.

The heart rate values were obtained continuously on both the Apple Watch and the commercial oximeters and were considered the value displayed after 15 s measurement, simultaneously on all devices.

### Statistical analysis

The sample size was calculated to identify a difference of 5% points between the devices. The level of significance was 0.05 and a power of 80%. A bilateral test was performed with paired samples. Shapiro–Wilk test was used to verify the normality of the data obtained.

The data was analyzed with graphs of frequency distribution and dispersion diagrams.

We used the Pearson test to assess correlations between groups (COPD and ILD patients and healthy volunteers). The Bland–Altman method was used to assess the accuracy and bias of the data. The values of the interclass correlation coefficient (two-way random-effects model) and One Way ANOVA were used as a basis for comparison between the groups.

The groups were paired and the level of significance was 0.05. The analyzes and graphs were performed using the *RStudio Team (Version 1.2.5019). RStudio: Integrated Development for R. RStudio, PBC, Boston, MA URL *http://www.rstudio.com/*.*

## Results

A total of 100 subjects were evaluated. The characteristics of the enrolled patients are shown in Table 1.

**Table 1:** Subject demographics.

Variable	Baseline disease			p
	COPD n (23)	ILD n (61)	Healthy n (16)	
Age	67.1	58.1	54.4	0.007
Gender (male)	39%	30%	46%	0.363
In use of oxygen	26%	15%	0%	< 0.001
**Oxygen saturation**				
Apple watch	95.1	95.8	97.4	0.025
Oximeter 1	94.8	94.8	97.4	< 0.001
Oximeter 2	94.2	92.5	96.4	0.007
**Heart rate**				
Apple watch	81.9	82.8	70.6	0.009
Oximeter 1	81.1	83.2	69.8	0.005
Oximeter 2	79.2	83.0	68.5	0.021

*ILD* Interstitial lung disease, *COPD* chronic obstructive pulmonary disease.

Among the 100 subjects, 23 had COPD, 61 ILD and 16 were healthy. The mean age of the groups was 67.1, 58.1, and 54.4, respectively.

Oxygen use was observed in 26% of patients with COPD and 15% of patients with ILD. When comparing the values of SpO_2_ and heart rate, they were significantly different in the healthy group when compared to the COPD and ILD groups in both commercial oximeters and Apple Watch devices.

In the comparative analyzes and correlations of the commercial oximeter devices for the parameters SpO_2_ and heart rate, we observed a strong relationship between them, r = 0.993 and 0.996, respectively. Therefore, in the other analyzes, we used the combination of the commercial oximeter devices data when compared to the Apple Watch device.

**Figure 1:**
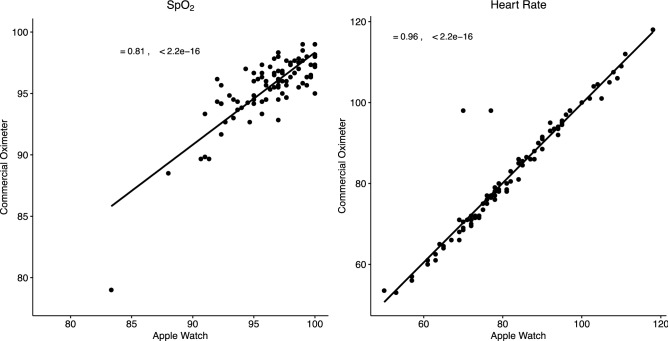
Correlation plots of SpO_2_ (r = 0.81) and heart rate (r = 0.966) of COPD and ILD patients and healthy volunteers.

We observed strong positive correlations between the Apple Watch device and commercial oximeters when evaluating heart rate measurements (r = 0.995, p < 0.001) and oximetry measurements (r = 0.81, p < 0.001) (Fig. 1). The two-way random-effects model for interclass correlation also shows good correlation for SpO_2_ (ICC = 0.896, p < 0.001) and heart rate (ICC = 0.963, p < 0.001).

**Figure 2:**
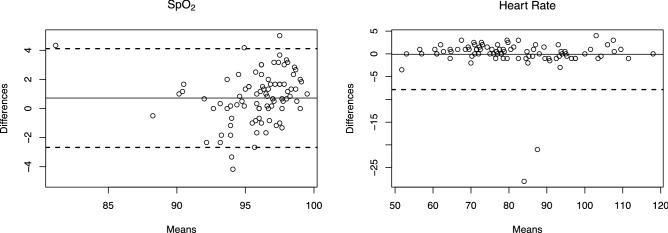
Bland–Altman plots of SpO_2_ and heart rate of COPD and ILD patients and healthy volunteers. Solid lines show the mean bias. Dashed bias represents upper and lower limits of agreement.

Bland–Altman plots were used to demonstrate the agreement between the SpO_2_ and Heart Rate measurements of the commercial oximeters and the Apple Watch devices. The absolute mean difference or bias of SpO_2_ between the devices was 0.8% and the limits of agreement ranged from − 2.7 to 4.1%. The mean difference or bias of Heart Rate between the devices was 0%, and the limits of agreement ranged from − 8 to 8% (Fig. 2).

**Figure 3:**
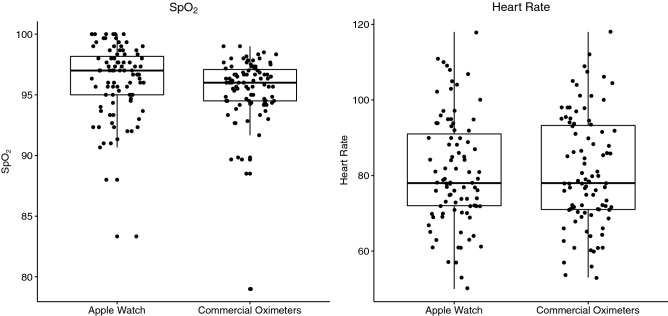
Comparison of Apple Watch and commercial oximeters devices of SpO_2_ and heart rate of COPD and ILD patients and healthy volunteers.

Based on the mean differences between the devices, the Apple Watch has a tendency of higher SpO_2_ values than commercial oximeters; however, heart rate measurements were similar in both devices (Fig. 3).

**Figure 4:**
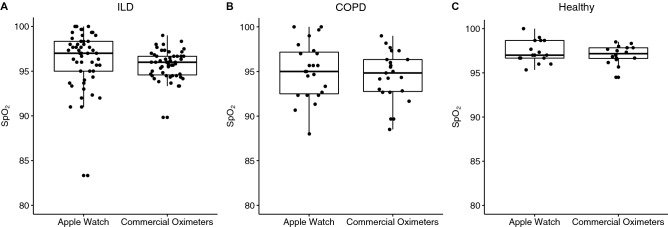
Comparison of Apple Watch and commercial oximeters devices of SpO_2_. (**A**) Interstitial Lung Disease (ILD), (**B**) Chronic Obstructive Pulmonary Disease (COPD) and (**C**) Healthy volunteers.

In the different groups, ILD, COPD, and healthy individuals, we did not observe significant differences between the Apple Watch and commercial oximeter devices for both parameters, SpO_2_ and heart rate (Fig. 4).

When we evaluated the different interferences that could alter the measurements in the devices using the interclass correlation coefficient (two-way random-effects model) and One Way ANOVA. We did not observe statistical difference in the evaluation of skin color, wrist circumference, presence of wrist hair, and enamel nail for SpO_2_ and heart rate measurements in Apple Watch or commercial oximeter devices (p > 0.05).

## Discussion

Chronic respiratory failure is usually the final stage of several respiratory diseases such as chronic obstructive pulmonary disease (COPD), pulmonary fibrosis, severe chest deformities, and acquired bronchiectasis. Patients living with hypoxemia and often hypercapnia, present important physical, psychological and social impairments with important deterioration in quality of life. In addition, these patients have repeated complications, with numerous hospitalizations^20^.

There is a wide variety of devices available that have the ability to manage and track medical parameters. These devices are capable of measuring blood pressure (BP), blood oxygen saturation, weight, temperature, and the number of daily steps performed, in addition to the electrocardiogram (ECG) record, allowing the patient to monitor his vital parameters in house.

The early detection of these parameters and possible complications are vitally important to trigger a medical service early or to monitor health. Videoconferences between doctors and patients can save patients time and money, especially in rural areas^21^.

Smartphone pulse oximetry applications can be simple for individuals to monitor their heart rate and SpO_2_ at home or remotely. Many patients who use household oxygen or who have chronic lung disease may find it useful and convenient to manage their daily activities by monitoring SpO_2_ and heart rate using smartphones or commercial oximeters. Thus, this study was carried out to assess whether the data obtained from these applications can be useful in patients with lung diseases.

Pilling and Cutaia^22^ demonstrated that oximeters are viable and accurate with an acceptable level of motion artifacts. Their results suggest ambulatory oximetry holds promise as a tool to monitor the adequacy of oxygen prescriptions in the outpatient with lung disease.

In this study, we evaluated the pulse oximetry technology, Apple Watch 6, in lung disease patients at risk of hypoxemia and compared two conventional commercial oximeter devices.

We observed strong correlations between these devices both in the evaluation of SpO_2_ and in the heart rate when we evaluated all groups unified and separated by ILD, COPD, and healthy individuals.

Tomlinson et al.^23^ assessed healthy pediatric subjects using smartphone apps, and Alexander et al.^24^ studied populations of healthy adults. Both studies reported that smartphone pulse oximeters were accurate. These data corroborate with our study that demonstrated that despite a tendency to present higher values, when compared to conventional oximetry devices, the Apple Watch device was accurate and similar.

In a study developed by Tayfur^25^ and collaborators comparing the use of smartphones in patients of different etiologies including a group of lung disease; the authors show that the values obtained by the smartphone were consistent with the reference devices and concluded that advances in technology may be improving the pulse oximetry of smartphones.

Conventional commercial oximeters have known limitations due to low perfusion, skin pigmentation, movement artifacts and the presence of nail polish^26,27^. Another specific condition, but an important clinical disease, is systemic sclerosis. Patients may develop hardening and tightening of the skin and connective tissues, including finger, which can impair proper reading of pulse oximeters^28^. In our study, three patients with systemic sclerosis were excluded due to inability to obtain data from the pulse oximeter. However, there was no problem with the Apple Watch reading. All of those problems could be minimized in oximeters using a smartwatch system.

In our study, we did not observe statistical differences between the SpO_2_ and heart rate in the Apple Watch or commercial oximeter devices measurements for skin color, wrist circumference, presence of wrist hair or enamel nail.

However, in a recent study published by the American Journal of Nursing indicated inaccuracies in oximetry in a group of black people. They highlight the need for a careful assessment of the signs and symptoms of this patient group and other clinical data in the treatment of COVID-19 and other critical diseases^29^.

Regarding cardiac parameters, constant monitoring can provide important clinical information. The variability of heart rate and systolic blood pressure provides information regarding autonomic control, as it quantitatively expresses the result of the action of the autonomic nervous system on the cardiovascular system. The smaller the heart rate fluctuation, the greater the risk of heart disease. In addition, this variability is an important prognostic factor for the onset of cardiac events in previously healthy individuals and in patients with heart disease^30^.

The use of constant monitoring with the watch can potentially bring benefits for the long-term control of these diseases by allowing the patient to manually adjust the oxygen flow values or even to develop some equipment integrated with the Apple Watch that adjusts automatically. In addition, all data that is already collected in smartwatches, such as movement and heart rate, can help determine the patient’s change in oxygen demand and the device can ask the patient to be in an adequate position to measure oxygen saturation if needed. Moreover, in the future, as the technology is validated for medical use, doctors can be promptly notified about a decrease in the patient oxygen saturation and take the necessary measures.

A point that we must emphasize in this study is that all measurements were carried out in a single timepoint with the patient positioned correctly and with the help of the researcher. Studies with continuous monitoring and during daily situations are still needed to confirm the possible use of data to guide medical treatment. The presence of a gyroscope in the smartwatch can help to determine the exact spatial position of wrist and whether the patient is walking or moving. This information can be used to decide the best time to measure during the day, so the patient is not moving. Moreover, as it can detect movement, a software is able to determine whether the measurement was obtained in a proper condition.

Other studies evaluating smartphone pulse oximeters in comparison with hospital-level digital probe oximeters and compared with blood oximetry are recommended. The validity of cheaper devices is also important in terms of public health, especially in developing countries. In addition, as smartphone technology continues to improve, studies to assess accuracy and reliability in various disease states are warranted.

## Conclusion

Our results indicate that Apple Watch 6 is a reliable way to obtain heart rate and SpO_2_ in patients with lung diseases under controlled conditions. The advance of smartwatch technology continues to improve and studies to assess accuracy and reliability in various types of disease should be carried out.
